# Self-Reported Frequency of Adding Salt to Food and Risk of Incident Chronic Kidney Disease

**DOI:** 10.1001/jamanetworkopen.2023.49930

**Published:** 2023-12-28

**Authors:** Rui Tang, Minghao Kou, Xuan Wang, Hao Ma, Xiang Li, Yoriko Heianza, Lu Qi

**Affiliations:** 1 Department of Epidemiology, School of Public Health and Tropical Medicine, Tulane University, New Orleans, Louisiana; 2 Department of Nutrition, Harvard T.H. Chan School of Public Health, Boston, Massachusetts

## Abstract

**Importance:**

The self-reported frequency of adding salt to foods could reflect a person’s long-term salt taste preference, and salt intake has been associated with increased risk of cardiovascular diseases (CVD). Whether self-reported adding of salt to foods is associated with increased risk of chronic kidney disease (CKD) remains unknown.

**Objective:**

To prospectively examine the association of self-reported frequency of adding salt to foods with incident CKD risk in a general population of adults.

**Design, Setting, and Participants:**

This population-based cohort study evaluated UK Biobank participants aged 37 to 73 years who were free of CKD at baseline. Participants were enrolled from 2006 to 2010 and prospectively followed up for disease diagnosis. Data were analyzed from October 2022 to April 2023.

**Exposure:**

Self-reported frequency of adding salt to foods, categorized into never or rarely, sometimes, usually, and always.

**Main Outcome and Measure:**

Incident CKD cases were defined by diagnostic codes. Hazard ratios (HRs) and 95% CIs were calculated by using Cox proportional hazards models. Models were adjusted for several potential confounders including age, sex, race and ethnicity, Townsend Deprivation Index, estimated glomerular filtration rate (eGFR), body mass index, (BMI), smoking status, alcohol drinking status, regular physical activity, high cholesterol, diabetes, CVD, hypertension, infectious disease, immune disease, and nephrotoxic drugs use at baseline.

**Results:**

Within a cohort of 465 288 individuals (mean [SD] age 56.32 [8.08] years; 255 102 female participants [54.83%]; 210 186 male participants [45.17%]), participants with higher self-reported frequency of adding salt to foods were more likely to have a higher BMI, higher Townsend Deprivation Index score, and diminished baseline eGFR compared with those who reported a lower frequency of adding salt to foods. Participants who added salt to their foods were also more likely than those who did not add salt to their foods to be current smokers and have diabetes or CVD at baseline. During a median (IQR) follow-up of 11.8 (1.4) years, 22 031 incident events of CKD were documented. Higher self-reported frequency of adding salt to foods was significantly associated with a higher CKD risk after adjustment for covariates. Compared with those who reported never or rarely adding salt to foods, those who reported sometimes adding salt to food (adjusted HR [aHR], 1.04; 95% CI, 1.00-1.07), those who reported usually adding salt to food (aHR, 1.07; 95% CI, 1.02-1.11), and those who reported always adding salt to food (aHR, 1.11; 95% CI, 1.05-1.18) had an increased risk of CKD (*P* for trend < .001). In addition, eGFR, BMI, and physical activity significantly modified the associations, which were more pronounced among participants with a higher eGFR, lower BMI, or lower level of physical activity.

**Conclusions and Relevance:**

In this cohort study of 465 288 individuals, a higher self-reported frequency of adding salt to foods was associated with a higher risk of CKD in the general population. These findings suggest that reducing the frequency of adding salt to foods at the table might be a valuable strategy to lower CKD risk in the general population.

## Key Points

**Question:**

Is self-reported frequency of adding salt to food associated with increased risk of chronic kidney disease (CKD) in the general population, and if so, is there any modification by other risk factors?

**Findings:**

In this cohort study involving 465 288 participants from the UK Biobank, a higher self-reported frequency of adding salt to foods was significantly associated with an increased risk of CKD. The associations were more pronounced among participants with a higher estimated glomerular filtration rate and lower body mass index or physical activity level.

**Meaning:**

These findings suggest that adding salt to foods is associated with increased risk of CKD in the general population, emphasizing the possible value of limiting discretionary salt to reduce CKD risk.

## Teaser

This cohort study examines whether frequency of adding salt to food as determined by self-report is associated with increased risk of chronic kidney disease (CKD).

## Introduction

Sodium intake is essential to human health because it serves various physiological functions, including maintaining fluid balance and nutrient absorption.^1^ The association of high sodium consumption with hypertension has been well established in previous studies.^2,3,4^ On one hand, hypertension is associated with increased risk of chronic kidney disease (CKD)^5,6^; on the other hand, hypertension often coexists with CKD, and shares several common risk associations, especially sodium intake.^7,8^ Although many studies to date have investigated the association of dietary sodium intake with the risk of CKD, most of them have only included participants who have already received a diagnosis of hypertension, diabetes, or other chronic conditions,^9,10,11,12^ whereas few studies have investigated the association of sodium intake with risk of CKD in the general population. Additionally, some previous studies^12,13^ investigating the association of dietary sodium intake with CKD have generated conflicting results, with either a decreased risk or no association reported. These studies^12,13^ relied on a single day’s urine collection or dietary survey to estimate the sodium intake, which is insufficient to assess an individual’s habitual consumption levels because sodium intake varies widely from day to day. Furthermore, several other methodological issues with sodium measurements exist.^14^ Therefore, research on the association of long-term dietary sodium intake with CKD risk is needed.

We recently found that higher self-reported frequency of adding salt to foods (usually at the table), a common eating behavior shaped by a person’s long-term preference for salty taste in foods and habitual salt intake, was associated with increased risk of cardiovascular diseases,^15^ premature mortality,^16^ and type 2 diabetes.^17^ Moreover, we found a graded association of self-reported frequency of adding salt to foods with concentration of estimated 24-hour sodium excretion.^16^ Of note, the most commonly used table salt contains 97% to 99% sodium chloride and may minimize the potential confounding effects due to associations with other dietary factors, particularly potassium. Therefore, self-reported adding of salt to foods presents a unique way to investigate the association of habitual sodium intake with CKD. However, to our knowledge, no study has explored whether the frequency of adding salt to food is associated with CKD risk in general population in a prospective setting. In this study, we analyzed the association of self-reported frequency of adding salt to foods with incident CKD among adults from the UK Biobank (UKB) study^18^ and explored association modification by estimated glomerular filtration rate (eGFR) and lifestyle factors.

## Methods

### Study Population

This cohort study was approved by the Tulane University Biomedical Committee institutional review board and followed the Strengthening the Reporting of Observational Studies in Epidemiology (STROBE) reporting guideline. The approval of UKB study protocol was received from the UK Northwest Multicenter Research Ethics Committee. The UKB is a national, population-based cohort study designed to improve the prevention, diagnosis, and treatment of various illnesses and promote health; the study design and methods have been presented previously.^18^ In brief, more than 500 000 participants aged 37 to 73 years were recruited across England, Scotland, and Wales between 2006 and 2010 at baseline. Those participants were living within 25 miles of 1 of the 22 UKB assessment centers and were registered within the UK National Health Service. Our initial study pool included data from 502 505 participants; we excluded 1126 participants who had incomplete data on the frequency of adding salt to foods at baseline and 36 091 participants who had prevalent CKD (eFigure in Supplement 1). Written informed consent was provided by all participants.

### Exposure Assessment

Information on salt intake and diet was collected through a touchscreen questionnaire. To assess the frequency of adding salt to foods at baseline, participants were asked “Do you add salt to your foods? (Do not include salt used in cooking).” Participants chose 1 answer from 5 options: (1) “never/rarely,” (2) “sometimes,” (3) “usually,” (4) “always,” and (5) “prefer not to answer.” Those who chose “prefer not to answer” were assigned as missing value and then excluded. To assess diet, participants were asked, “Have you made any major changes to your diet in the last 5 years?” Participants chose 1 answer from the following 4 options: (1) “no,” (2) “yes, because of illness,” (3) “yes, because of other reasons,” and (4) “prefer not to answer.”

### Ascertainment of CKD

Incident CKD cases were defined by *International Statistical Classification of Diseases and Related Health Problems, Tenth Revision (ICD-10)* codes and *Office of Population Census and Surveys Classification of Interventions and Procedures, version 4 *(OPCS-4) codes, which can be found in the eMethods in Supplement 1. Hospital inpatient records were collected from linkage with the Hospital Episode Statistics (England), Scottish Morbidity Records (Scotland), and Patient Episode Database (Wales). Deaths of patients with CKD were obtained by linking to the death registry. Follow-up time was calculated from the date of baseline to diagnosis of CKD, death, or the censoring date (May 2021), whichever occurred first. Detailed information on the ascertainment of outcomes is available online.^19^ Prevalent CKD was additionally defined as an eGFR below 60 mL/min/1.73 m^2^ or albuminuria above 3 mg/mmol at baseline.

### Covariates Assessment

Potential confounders included age, sex, race and ethnicity, Townsend Deprivation Index^20^ (a composite measure of deprivation based on unemployment, noncar ownership, nonhome ownership, and household overcrowding, with a negative value representing higher socioeconomic status), smoking status (never, previous, and current), drinking status (never, previous, and current), and physical activity level. Race and ethnicity were determined via self-report from baseline and categories included Asian, Black, Chinese, White, multiracial, and other (defined as any other race or ethnicity not otherwise specified); race and ethnicity were included to understand potential racial and ethnic disparities in health outcomes. Patients were assessed at baseline using a touch-screen questionnaire. Regular physical activity was defined as 150 minutes or more per week of moderate intensity activity, 75 minutes or more per week of vigorous activity, or an equivalent combination per week. Standing height was assessed by the Seca 202 height measure (Seca). Weight and body composition were measured by the BC-418MA body composition analyzer (Tanita). Body mass index (BMI) was calculated as weight in kilograms divided by height in meters squared at the initial visit in the assessment center. High cholesterol was defined by self-reported history of high cholesterol or taking cholesterol lowering medications. A hypothesis-driven dietary pattern was generated to reflect the overall diet using 5 well-known heart health-related dietary components.^21,22,23,24^ Definitions and variables used for dietary components are shown in eTable 1 in Supplement 1. Blood samples were collected at baseline, and eGFR was calculated using the new creatinine and cystatin C based equations (without race) published in 2021.^25^ Assessment of other covariates (ie, history of hypertension, diabetes, cardiovascular disease [CVD], infectious disease, immune disease, and nephrotoxic drugs use) can be found in the eMethods in Supplement 1.

### Statistical Analyses

Hazard ratios (HRs) and 95% CIs were calculated by using Cox proportional hazards models with the follow-up time (ie, calendar time) as the time scale to estimate the associations of the self-reported frequency of adding salt to foods with risk of CKD. Then the multinomial variable indicating the frequency of adding salt to foods was treated as an ordinal variable to test for linear trends to obtain *P*-trend values. The proportional hazards assumption was tested using the Kaplan-Meier and Schoenfeld residuals methods; no violation of the assumption was observed (eTable 2 in Supplement 1). We adjusted several potential confounders in these models, including age, sex, race and ethnicity, Townsend Deprivation Index, eGFR, BMI, smoking status, alcohol drinking status, regular physical activity, high cholesterol, diabetes, CVD, hypertension, infectious disease, immune disease, and nephrotoxic drugs use at baseline. Missing data for continuous covariates was addressed with mean values, and categorical covariates were addressed with a missing indicator category.

We also performed stratified analyses by following factors: age (<60 years or ≥60 years), sex (male or female), race and ethnicity (White or Asian, Black, Chinese, multiracial and other), BMI (18.5-24.9, 25-29.9, and ≥30), Townsend Deprivation Index (quintile 1, quintiles 2-4, and quintile 5), eGFR (≥90 mL/min/1.73 m^2^ or 60 ≤ eGFR < 90 mL/min/1.73 m^2^), smoking (noncurrent or current), drinking (noncurrent or current), regular physical activity (no or yes), high cholesterol (no or yes), diet score (<median or ≥ median), baseline diabetes (no or yes), and baseline hypertension (no or yes). To assess interactions between the frequency of adding salt to foods and these factors, interaction terms were added to the original Cox models and the Wald test was performed.

We conducted several sensitivity analyses to test the robustness of our findings. First, we repeated the main analyses after excluding the individuals with hypertension at baseline. Second, we performed the main analyses after excluding the individuals with diabetes or CVD at baseline. Third, we performed the main analyses after excluding the individuals with acquired CKD within 12 months of recruitment. Fourth, we assessed whether the results would change after excluding participants who changed their diet in the last 5 years. Fifth, we further adjusted diet score to test whether diet score mediates the association of the self-reported frequency of adding salt to foods with the risk of CKD. Finally, we performed another sensitivity analysis to investigate the association of the self-reported frequency of adding salt to foods with risk of CKD by eGFR and by excluding smokers.

All statistical analyses were conducted using SAS statistical software version 9.4 (SAS Institute), and a 2-sided *P* < .05 was set as the threshold for statistical significance. Data were analyzed from October 2022 to April 2023.

## Results

Among the 465 288 participants in the cohort (mean [SD] age 56.32 [8.08] years; 255 102 female participants [54.83%]; 210 186 male participants [45.17%]), individuals with higher self-reported frequency of adding salt to foods displayed distinctive demographic and health profiles compared with their counterparts with lower frequency of adding salt to foods; they were predominantly female, Asian, Black, Chinese or multiracial, and had a higher BMI, higher Townsend Deprivation Index, and a decreased baseline eGFR. These individuals were also more likely to be current smokers, less likely to be current drinkers or engage in regular physical exercise, and generally exhibited a lower diet score. In addition, a higher prevalence of diabetes or CVD at baseline was observed among these participants (Table 1).

**Table 1.** Baseline Characteristics According to the Self-Reported Frequency of Adding Salt to Foods

Characteristic	Participants, No. (%) (N = 465 288)			
	Never or rarely add salt to food (n =258 531)	Sometimes add salt to food (n = 130 435)	Usually add salt to food (n = 53 964)	Always add salt to food (n = 22 358)
Age, mean (SD), y	56.33 (8.07)	56.22 (8.09)	56.79 (8.02)	55.69 (8.25)
Sex				
Female	145 829 (56.41)	71 008 (54.44)	26 664 (49.41)	11 601 (51.89)
Male	112 702 (43.59)	59 427 (45.56)	27 300 (50.59)	10 757 (48.11)
Race and ethnicity				
Asian	4041 (1.56)	3352 (2.57)	1538 (2.85)	1178 (5.27)
Black	3288 (1.27)	1538 (1.84)	800 (1.48)	764 (3.42)
Chinese	1708 (0.66)	800 (1.06)	562 (1.04)	449 (2.01)
White	247 298 (95.66)	122 054 (93.57)	50 503 (93.59)	19 645 (87.87)
Multiracial^a^	1307 (0.51)	838 (0.64)	370 (0.69)	220 (0.98)
Other^b^	889 (0.34)	764 (0.32)	449 (0.35)	102 (0.46)
Body mass index, mean (SD)^c^	27.07 (4.64)	27.52 (4.72)	27.71 (4.72)	27.92 (5.03)
Townsend Deprivation Index score, mean (SD)	−1.52 (2.97)	−1.25 (3.09)	−1.11 (3.15)	−0.23 (3.49)
Baseline estimated glomerular filtration rate, mean (SD) mL/min/1.73 m^2^	97.23 (12.53)	96.71 (12.70)	95.86 (12.78)	95.99 (13.16)
Current smoking	20 355 (7.87)	14 507 (11.12)	8076 (14.97)	5241 (23.44)
Current drinking	238 077 (92.09)	121 079 (92.83)	49 951 (92.56)	19 588 (87.61)
Regular physical activity	159 406 (61.66)	79 322 (60.81)	31 907 (59.13)	12 459 (55.73)
High cholesterol	41 943 (16.22)	20 206 (15.49)	8716 (16.15)	3456 (15.46)
Diabetes	6295 (2.27)	3392 (2.43)	1354 (2.35)	603 (2.50)
Cardiovascular disease	12 187 (4.71)	5834 (4.47)	2619 (4.85)	1278 (5.72)
Diet score, mean (SD)^d^	3.05 (1.44)	2.77 (1.44)	2.53 (1.44)	2.26 (1.42)

^a^ The multiracial category combined the responses of the UK Biobank race and ethnicity questions of “mixed,” “White and Black Caribbean,” “White and Black African,” “White and Asian,” and “any other mixed backgrounds.”

^b^ Other was defined as any other race or ethnicity not otherwise specified.

^c^ Body mass index was calculated as weight in kilograms divided by height in meters squared.

^d^ Diet score included intake of vegetables, fruits, fish, whole grains, processed meat, and red meat.

The associations of the self-reported frequency of adding salt to foods with the risk of CKD are shown in Table 2. During a median (IQR) follow up of 11.8 (1.4) years, we documented 22 031 incident events of CKD in this study. In the sex-adjusted and age-adjusted model, higher self-reported frequency of adding salt to foods was significantly associated with a higher risk of CKD for those who reported sometimes adding salt to food (adjusted HR, [aHR] 1.07; 95% CI, 1.04-1.10), those who reported usually adding salt to food (aHR, 1.12; 95% CI, 1.07-1.16), and those who reported always adding salt to food (aHR, 1.29; 95% CI, 1.22-1.37) compared with those who reported never or rarely adding salt to food (*P *for trend < .001). After further adjusting for race and ethnicity, BMI, Townsend Deprivation Index, smoking, drinking, regular physical activity, high cholesterol, CVD, and diabetes at baseline, the association was attenuated but still significant for those who reported sometimes adding salt to food (aHR, 1.04; 95 % CI, 1.00-1.07), those who reported usually adding salt to food (aHR, 1.07; 95% CI, 1.02-1.11), and those who reported always adding salt to food (aHR, 1.11; 95% CI, 1.05-1.18) compared with those who reported never or rarely adding salt to food (*P *for trend < .001). Moreover, after further adjusting for eGFR, higher self-reported frequency of adding salt to foods was still significantly associated with a higher risk of CKD for those who reported sometimes adding salt to food (aHR, 1.02; 95% CI, 0.98-1.05), those who reported usually adding salt to food (aHR, 1.05; 95% CI, 1.01-1.10), and those who reported always adding salt to food (aHR, 1.09; 95% CI, 1.03-1.16) compared with those who reported never or rarely adding salt to food (*P *for trend = .001). In addition, upon further controlling for hypertension, infectious disease, immune disease, and nephrotoxic drugs at baseline, the association was still significant for those who reported sometimes adding salt to food (aHR, 1.02; 95% CI, 0.99-1.06), those who reported usually adding salt to food (aHR, 1.05; 95% CI, 1.01-1.10), and those who reported always adding salt to food (aHR, 1.06; 95% CI, 1.02-1.12) compared with those who reported never or rarely adding salt to food (*P *for trend = .004).

**Table 2.** Self-Reported Frequency of Adding Salt to Foods and Risk of Chronic Kidney Disease

Model	Frequency of adding salt to food, HR (95% CI)				*P* for trend
	Never or rarely	Sometimes	Usually	Always	
No. of chronic kidney disease cases/total No.	11 590/258 531	6300/130 435	2881/53 964	1260/22 358	
Sex and age adjusted	1 [Reference]	1.07 (1.04-1.10)	1.12 (1.07-1.16)	1.29 (1.22-1.37)	<.001
Multivariable adjusted^a^	1 [Reference]	1.04 (1.00-1.07)	1.07 (1.02-1.11)	1.11 (1.05-1.18)	<.001
Multivariable adjusted and adjusted for estimated glomerular filtration rate^a^	1 [Reference]	1.02 (0.98-1.05)	1.05 (1.01-1.10)	1.09 (1.03-1.16)	.001
Multivariable adjusted and adjusted for hypertension, infectious diseases, immune diseases, and nephrotoxic drugs at baseline^a^	1 [Reference]	1.02 (0.99-1.06)	1.05 (1.01-1.10)	1.06 (1.02-1.12)	.004

Abbreviation: HR, hazard ratio.

^a^ Adjusted for sex, age, race and ethnicity, body mass index, Townsend Deprivation Index, smoking, drinking, regular physical activity, high cholesterol, cardiovascular diseases, and diabetes at baseline.

**Figure 1.**
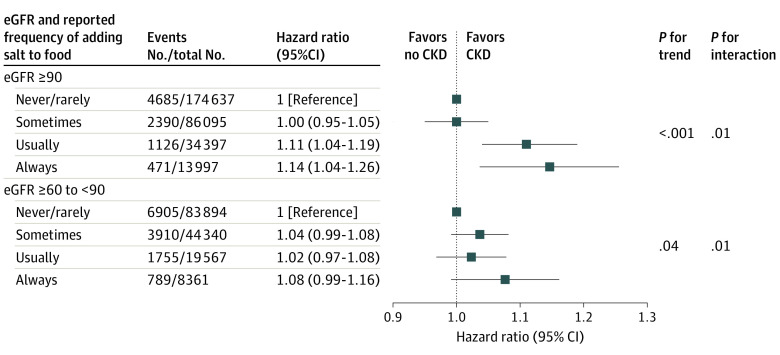
Interaction Between Estimated Glomerular Filtration Rate (eGFR) and the Association of Self-Reported Frequency of Adding Salt to Foods With Risk of Chronic Kidney Disease (CKD) Results were adjusted for sex, age, race and ethnicity, body mass index, Townsend Deprivation Index, smoking, drinking, regular physical activity, high cholesterol, cardiovascular diseases, and diabetes at baseline.

**Figure 2.**
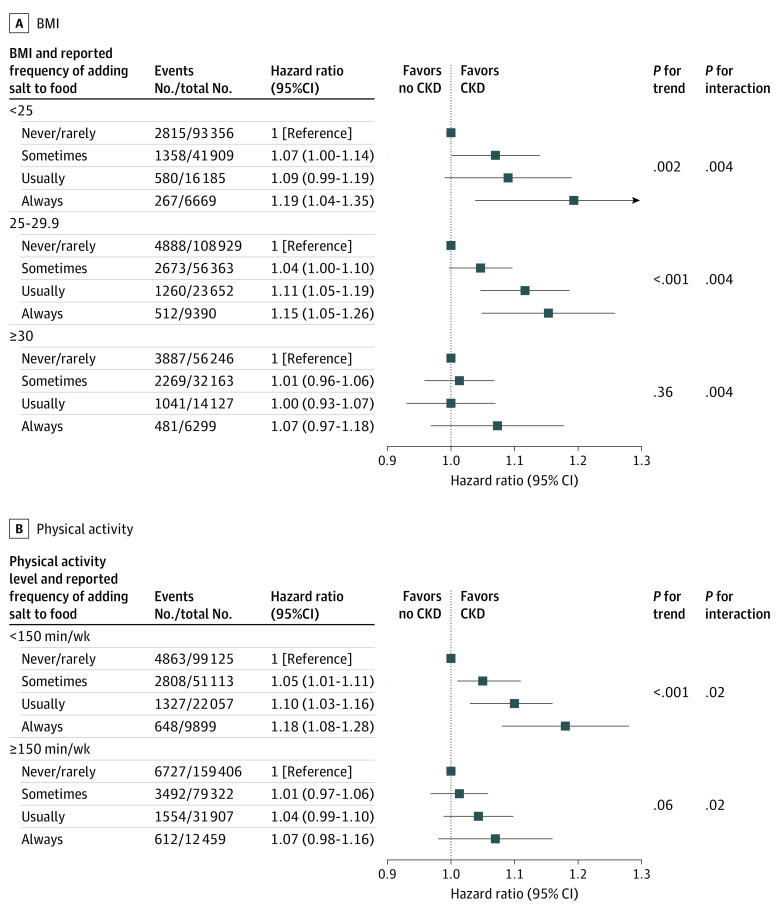
Interaction Between Body Mass Index (BMI) or Regular Physical Activity and the Association of Self-Reported Frequency of Adding Salt to Foods With Risk of Chronic Kidney Disease (CKD) Results were adjusted for sex, age, race and ethnicity, BMI, Townsend Deprivation Index, smoking, drinking, regular physical activity, high cholesterol, cardiovascular diseases, and diabetes at baseline. BMI was calculated as weight in kilograms divided by height in meters squared.

We also conducted stratified analyses according to eGFR and other potential factors associated with increased risk of CKD including age, sex, race and ethnicity, BMI, Townsend Deprivation Index, smoking, drinking, physical activity level, high cholesterol, diet score, baseline diabetes, and baseline hypertension (Table 3) to evaluate whether the covariates modified the association of the self-reported frequency of adding salt to foods with risk of CKD. We found a positive association of adding salt to foods with CKD appeared to be increased with increasing level of eGFR. Higher self-reported frequency of adding salt to foods was both significantly associated with a higher risk of CKD in participants with eGFR of 90 mL/min/1.73 m^2^ or higher and eGFR greater than or equal to 60 mL/min/1.73 m^2^ and less than 90 mL/min/1.73 m^2^ (Figure 1). We observed that the positive association of adding salt to foods with CKD was attenuated with increased BMI and level of physical activity, as well as the presence of high cholesterol. Higher self-reported frequency of adding salt to foods was significantly associated with higher risk of CKD in participants with normal weight and in participants who were overweight, whereas the association was not significant for participants who were obese (BMI ≥ 30) (Figure 2A). Higher self-reported frequency of adding salt to foods was significantly associated with higher risk of CKD in participants without regular physical activity (Figure 2B). Higher self-reported frequency of adding salt to foods was significantly associated with higher risk of CKD in participants without high cholesterol (aHR for those who reported sometimes adding salt to food; 1.04 [95% CI, 1.00-1.08]; aHR for those who reported usually adding salt to food; 1.07 [95% CI, 1.02-1.12]; aHR for those who reported always adding salt to food, 1.19 [95% CI, 1.10-1.28]; *P* for trend < .001) (Table 3). We did not find significant interactions between other factors and the association of the self-reported frequency of adding salt to foods with the risk of CKD.

**Table 3.** Stratified Analyses for the Association of Self-Reported Frequency of Adding Salt to Food and Risk of Chronic Kidney Disease

Subgroups	Frequency of adding salt to food, HR (95% CI)^a^				*P* for trend	*P* for interaction
	Never or rarely	Sometimes	Usually	Always		
Age, y						
<60	1 [Reference]	1.03 (0.98-1.08)	1.10 (1.03-1.18)	1.12 (1.02-1.24)	.001	.76
≥60	1 [Reference]	1.04 (1.00-1.08)	1.07 (1.01-1.22)	1.12 (1.04-1.21)	<.001	
Sex						
Female	1 [Reference]	1.00 (0.95-1.04)	1.03 (0.97-1.10)	1.20 (1.10-1.27)	.008	.91
Male	1 [Reference]	1.06 (1.02-1.11)	1.10 (1.04-1.15)	1.10 (1.01-1.18)	<.001	
Race and ethnicity						
Asian, Black, Chinese, multiracial,^b^ and other^c^	1 [Reference]	1.08 (0.94-1.23)	0.98 (0.81-1.18)	1.04 (0.85-1.28)	.76	.52
White	1 [Reference]	1.05 (1.01-1.10)	1.05 (0.99-1.11)	1.17 (1.08-1.27)	<.001	
Townsend Deprivation Index score, quintile						
1 (Low)	1 [Reference]	1.08 (1.01-1.16)	1.09 (1.00-1.20)	1.18 (1.02-1.37)	.003	.07
2-4 (Intermediate)	1 [Reference]	1.02 (0.98-1.06)	1.05 (1.00-1.10)	1.10 (1.02-1.18)	.006	
5 (High)	1 [Reference]	1.03 (0.96-1.11)	1.10 (1.00-1.21)	1.28 (1.10-1.49)	.001	
Smoking status						
Noncurrent	1 [Reference]	1.04 (1.00-1.07)	1.07 (1.02-1.12)	1.15 (1.07-1.22)	<.001	.07
Current	1 [Reference]	1.04 (0.94-1.14)	1.07 (0.96-1.20)	1.07 (0.93-1.22)	.19	
Drinking status						
Noncurrent	1 [Reference]	0.94 (0.85-1.03)	1.01 (0.88-1.14)	1.02 (0.87-1.19)	.99	.21
Current	1 [Reference]	1.04 (1.01-1.08)	1.07 (1.03-1.12)	1.13 (1.06-1.21)	<.001	
High cholesterol						
No	1 [Reference]	1.04 (1.00-1.08)	1.07 (1.02-1.12)	1.19 (1.10-1.28)	<.001	.007
Yes	1 [Reference]	1.01 (0.96-1.07)	1.06 (0.99-1.14)	0.98 (0.88-1.10)	.38	
Diet score^d^						
<Median	1 [Reference]	1.03 (0.98-1.08)	1.03 (0.97-1.10)	1.07 (0.98-1.16)	.08	.58
≥Median	1 [Reference]	1.01 (0.96-1.05)	1.05 (0.99-1.11)	1.13 (1.03-1.24)	.02	
Baseline diabetes						
No	1 [Reference]	1.03 (1.00-1.06)	1.06 (1.02-1.11)	1.13 (1.06-1.20)	<.001	.39
Yes	1 [Reference]	1.06 (0.95-1.18)	1.08 (0.93-1.25)	1.04 (0.84-1.30)	.32	
Baseline hypertension^e^						
No	1 [Reference]	1.04 (0.99-1.08)	1.08 (1.02-1.14)	1.16 (1.07-1.25)	<.001	.46
Yes	1 [Reference]	1.06 (1.01-1.11)	1.10 (1.04-1.18)	1.14 (1.04-1.24)	<.001	

Abbreviation: HR, hazard ratio.

^a^ Models were adjusted for sex, age, race and ethnicity, BMI, Townsend Deprivation Index, smoking, drinking, regular physical activity, high cholesterol, cardiovascular diseases, and diabetes at baseline.

^b^ The multiracial category combined the responses of the UK Biobank race and ethnicity questions of “mixed,” “White and Black Caribbean,” “White and Black African,” “White and Asian,” and “any other mixed backgrounds.”

^c^ Other was defined as any other race or ethnicity not otherwise specified.

^d^ Results were further adjusted for diet score.

^e^ Results were further adjusted for hypertension.

Our results were robust in several sensitivity analyses, which are presented in eTable 3 in Supplement 1. Excluding participants who had hypertension at baseline yielded similar results with main analyses. Further, when we performed the main analysis excluding participants who had diabetes or CVD at baseline, the risk estimates were still similar. Moreover, in comparison with the individuals with acquired CKD after 3 months of recruitment, the HRs were slightly attenuated (HR for those who reported sometimes adding salt to food, 1.03 [95% CI, 1.00-1.06]; HR for those who reported usually adding salt to food, 1.06 [95% CI, 1.02-1.10]; HR for those who reported always adding salt to food, 1.12 [95% CI, 1.05-1.19]; *P* for trend < .001). In addition, these results did not significantly change after excluding participants who changed their diet in the last 5 years. The results remained consistent after further adjusting for diet score. Finally, after the exclusion of individuals with a history of smoking, the interaction between eGFR and the association of frequency of adding salt to foods with CKD was no longer significant (eTable 4 in Supplement 1).

## Discussion

In this prospective cohort study, the association of the self-reported frequency of adding salt to foods with the risk of CKD was evaluated in individuals free of CKD at baseline. We observed that higher self-reported frequency of adding salt to foods was significantly associated with a higher risk of incident CKD independent of socioeconomic and lifestyle factors, as well as other traditional factors associated with increased risk of CKD. In addition, we found that the positive association was increased among participants with a normal or higher eGFR, whereas the positive association appeared to be attenuated among participants with obesity and those who were physically active.

Our findings of the adverse association of frequent adding of salt to foods with CKD risk are supported by several diet intervention trials.^26,27,28^ In addition, the findings in our study are also consistent with several studies^29,30^ using 24-hour urine samples, in which positive associations of sodium intake with kidney function decline were observed.

Very few studies have investigated the association of long-term dietary sodium intake with the development of CKD in the general population. In addition, previous studies^10,11,12,31^ investigating the association of dietary sodium intake with CKD have generated conflicting results, finding either no association or a spuriously increasing risk of CKD at lower level of sodium intake. The amount of salt consumed fluctuates greatly from day to day. Using a single day’s measurement to determine an individual’s usual sodium consumption is a major limitation in earlier studies, likely resulting in significant random errors in sodium assessment and substantially confounding the association of sodium intake with health outcomes.^14,32,33^ In this study, we offered a novel viewpoint to examine the association of salt usage behaviors with CKD. Even though the frequency of adding salt to foods is not a source of quantitative data on overall sodium intake, the graded association of the frequency of adding salt to foods with levels of concentration of estimated 24-hour sodium excretion represents a person’s long-term preference for salt taste, and it is less likely to be impacted by the daily variations in sodium intake.^16,34,35^ Our recent research^15,16,17^ and additional study^36^ have demonstrated the validity of self-reported adding salt to foods as a good indicator for long-term sodium intake. Furthermore, the commonly used table salt has between 97% and 99% sodium chloride, which can minimize other dietary factors’ (eg, potassium) potential confounding effects. Therefore, adding salt to foods is a unique method to assess habitual sodium intake compared with previous measures.

The subgroup analyses showed that the association of sodium intake with risk of CKD was attenuated in participants with a lower eGFR than those with a higher eGFR. This observation should be interpreted with caution. Notably, smoking has been associated with a lower eGFR^37,38^ but a higher liking for salty taste.^39^ After excluding smokers (eTable 4 in Supplement 1), the observed significant interaction between eGFR and the self-reported frequency of adding salt to foods was eliminated, indicating that the interaction between eGFR and the association of self-reported frequency of adding salt to foods with the risk of CKD might be partly mediated by smoking.

Intriguingly, we found that the positive associations of the self-reported frequency of adding salt to foods with the risk of CKD appeared to be attenuated with a higher BMI. Similar interaction patterns were observed in our previous studies,^16,36^ in which the positive association of the frequency of adding salt to foods with the risk of premature mortality was attenuated with increased BMI. Moreover, an epidemiological study^40^ observed that salt intake was significantly associated with systolic blood pressure among individuals with typical weight compared with individuals who were overweight and obese. Another possible explanation is that obesity may mask the association of sodium intake with risk of CKD, which was also observed in a prior study.^41^ The high proportion of individuals who were overweight or obese in this population could be the biggest contributor to masking associations. We also observed the association of sodium intake with CKD was lessened in participants with regular physical activity compared with those who were less physically active, suggesting optimal physical activity might lower the adverse association of high preference for salt with CKD. This finding is supported by prior studies^42,43^ that reported increased physical activity was associated with better CKD outcomes.

There are several biological mechanisms underlying the positive association of habitually high sodium intake with increased risk of CKD. High sodium intake is associated with aldosterone activation, extracellular fluid volume, renin-angiotensin-aldosterone system disturbances, failure of normal autoregulation of the peripheral vasculature,^44^ increased oxidative stress,^45^ proinflammatory cytokines,^46^ intrarenal angiotensin II,^47^ as well as increased arterial stiffness and/or endothelial dysfunction,^48^ all of which are associated with CKD. Further studies are warranted to explore the biological mechanisms of the association of sodium intake with risk of CKD.

To our knowledge, this is the first study indicating that higher self-reported frequency of adding salt to foods is associated with a higher CKD risk in the general population. The strengths of this study include the prospective design, large sample size, extensive information of covariates, the unique methods for investigating long-term habitual sodium intake, and the consistent results in sensitivity analyses.

### Limitations

Several potential limitations of our study should be carefully considered. First, the self-reported frequency of adding salt to foods was unable to provide precise quantitative data on dietary salt consumption and might be subject to information bias; however, previous studies^34,35^ have shown the validity of this variable. Second, we were unable to rule out the possibility that high frequency of adding salt to foods was a marker of an unhealthy lifestyle. However, we have carefully adjusted for lifestyle factors and the subgroup analyses suggested that the positive association of the frequency of adding salt to foods with risk of CKD was consistent across the subgroups of lifestyle factors. Third, there might be residual confounding. Fourth, the participants in the UKB study were mainly of European descent; it is unclear whether our findings could be applied to other populations and, thus, future studies are needed in other populations, particularly in subpopulations with unique dietary habits. Fifth, data regarding the frequency of adding salt to foods was collected only at the baseline of our study; therefore, we did not account for any changes in participants’ salt taste preferences over the course of the follow-up. Future research could investigate how alterations in preference for saltiness are associated with subsequent CKD risk.

## Conclusions

In conclusion, our study indicates that the higher self-reported frequency of adding salt to foods is significantly associated with a higher risk of CKD. These findings support the reduction of adding salt to foods as a potential intervention strategy for CKD prevention. Post hoc analysis or follow-up studies to clinical trials are necessary to validate these findings.

## Notes

Supplement 1. **eFigure.** Flow Chart
eMethods.
**eTable 1.** Definition of Diet Score in UK Biobank **eTable 2.** Proportional Hazard Assumption P-Values and Kaplan-Meier Plots **eTable 3.** Sensitivity Analyses for Association Between Self-Reported Frequency of Adding Salt to Food and Hazard of Chronic Kidney Disease **eTable 4.** The Association Between the Self-Reported Frequency of Adding Salt to Foods and Hazard of Chronic Kidney Disease by eGFR Levels (After Excluding Ever Smokers)
eReference

Supplement 2.
Data Sharing Statement
