# Supplementary material for: Self-Reported Frequency of Adding Salt to Food and Risk of Incident Chronic Kidney Disease
Source: JAMA Netw Open. 2023 Dec 28;6(12):e2349930. doi: 10.1001/jamanetworkopen.2023.49930 (PMC10755616; doi:10.1001/jamanetworkopen.2023.49930)
Supplement: Supplement 1. — eFigure. Flow Chart eMethods. eTable 1. Definition of Diet Score in UK Biobank eTable 2. Proportional Hazard Assumption P-Values and Kaplan-Meier Plots eTable 3. Sensitivity Analyses for Association Between Self-Reported Frequency of Adding Salt to Food and Hazard of Chronic Kidney Disease eTable 4. The Association Between the Self-Reported Frequency of Adding Salt to Foods and Hazard of Chronic Kidney Disease by eGFR Levels (After Excluding Ever Smokers) eReference [file jamanetwopen-e2349930-s001.pdf]

Supplemental Online Content

Tang R, Kou M, Wang X, et al. Self reported frequency of adding salt to food and risk of incident chronic kidney disease. *JAMA Netw. Open*. 2024;7(1):e2349930. doi:10.1001/jamanetworkopen.2023.49930

eFigure. Flow Chart

eMethods

eTable 1. Definition of Diet Score in UK Biobank

eTable 2. Proportional Hazard Assumption *P*-Values and Kaplan-Meier Plots

eTable 3. Sensitivity Analyses for Association Between Self-Reported Frequency of Adding Salt to Food and Hazard of Chronic Kidney Disease

eTable 4. The Association Between the Self-Reported Frequency of Adding Salt to Foods and Hazard of Chronic Kidney Disease by eGFR Levels (After Excluding Ever Smokers)

eReference

This supplemental material has been provided by the authors to give readers additional information about their work.

© 2023 Tang R et al. *JAMA Network Open.*

eFigure. Flow Chart

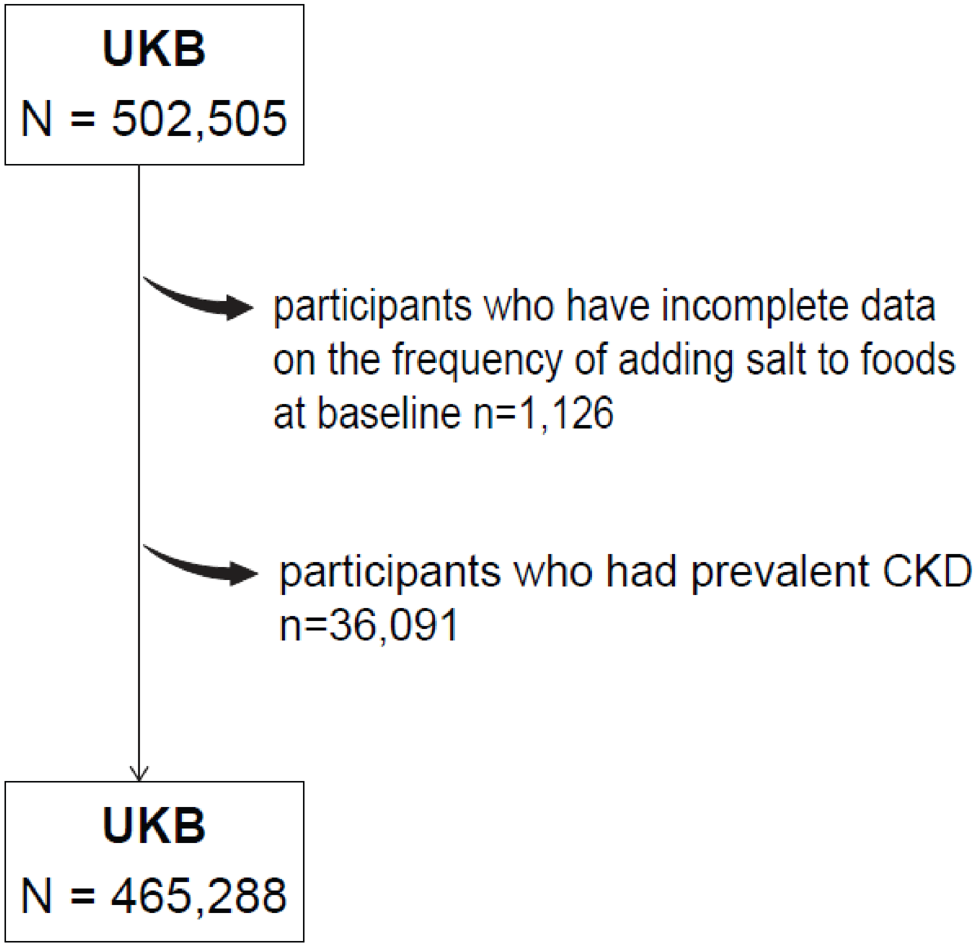

© 2023 Tang R et al. *JAMA Network Open.*

eMethods

Ascertainment of CKD

Primary care data, hospital inpatient data, and death register records were identified on the basis of ICD codes (N03, N06, N08, N11, N12, N13, N14, N15, N16, N18, N19, N20, and N21). Indications of renal replacement therapy cases were identified using hospital inpatient data based on ICD-10 codes (E85.3, N16.5, Q60.1, T82.4, T86.1, Y60.2, Y61.2, Y62.2, Y84.1, Z49.0, Z49.1, Z49.2, Z94.0, and Z99.2) and OPCS-4 codes (L74.1, L74.2, L74.3, L74.4, L74.5, L74.6, L74.8, L74.9, M01.2, M01.3, M01.4, M01.5, M01.8, M01.9, M02.3, M08.4, M17.2, M17.4, M17.8, M17.9, X40.1, X40.2, X40.3, X40.4, X40.5, X40.6, X40.7, X40.8, X40.9, X41.1, X41.2, X41.8, X41.9, X42.1, X42.8, X42.9, and X43.1) [1].

History of hypertension

Hypertension is defined as: (1) self-reported hypertension in the touchscreen and verbal interview (field 20002: 1065; field 6150: 4); (2) measured systolic blood pressure ≥ 140 or diastolic blood pressure 90 mmHg twice at recruitment; (3) taking antihypertensive medication (filed 6177: 2 and 6153:2); (4) diagnosis indicative of hypertension in primary care and hospital inpatient data (ICD-10: I11–I15; ICD-9: 401–405).

History of diabetes

Diabetes include Type 1 and Type 2 diabetes, which are defined according to the ICD-10 codes: E10 and E10.

History of CVD

CVD outcomes were collected via medical history and linkage to data on hospital admissions, questionnaires, and the death register data. IHD is defined by ICD-10 code I21-I25; Stroke is defined by ICD-10 code I60-I64; Heart failure is defined by ICD-10 code I110, I113 and I50.

History of infection diseases

Infection disease is defined as:
ICD-9 code: "0020", "0030", "0031", "0039", "0043", "0080", "00840", "00849", "0084", "0085", "0050", "0059", "0063", "0069", "0071", "0072", "0078", "0086", "0088", "0090", "0091", "0092", "0093", "01192", "01199", "0130", "3204", "015", "0160", "01619", "01699", "0172", "01729", "0170", "01789", "0239", "0270", "0339", "0360", "3205", "0362", "0382", "0380", "0381", "0384", "0388", "0389", "0359", "0418", "0419", "0913", "0979", "0988", "0980", "0992", "1310", "0541", "1021", "1048", "1049", "0739", "3232", "0459", "0489", "0479", "0490", "3216", "3217", "0540", "0549", "0543", "0544", "0529", "0531", "0532", "0539", "0559", "0781", "0780", "0701", "0703", "0705", "0709", "0720", "0721", "3215", "0723", "0729", "0759", "0741", "1101", "1104", "1105", "1108", "1120", "1121", "1128", "1129", "1173", "0840", "0841", "0846", "1309", "1363", "1220", "1289", "0412", "0410", "0411", "0414", "0415", "0417", "0792", "0799", "1368", "1369", "0788", "3202", "3207", "3229", "3234", "3236", "3239", "3240", "3731", "3732", "3730", "3735", "3738", "3739", "3760", "3600", "3801", "38019", "3820", "3830", "4210", "4219", "4610", "4611", "4618", "4619", "4629", "0340", "4639", "4640", "4641", "4642", "4643", "4871", "4878", "4809", "4819", "4824", "4828", "4829", "4839", "4848", "4859", "4869", "4660", "4759", "5109", "5210", "5220", "5224", "5226", "5227", "5225", "5233", "5234", "5283", "5400", "5401", "5409", "5669", "5672", "5720", "5721", "6849", "6800", "6802", "6806", "6810", "6811", "6819", "6823",

© 2023 Tang R et al. *JAMA Network Open.*

"6824", "6826", "6827", "6820", "6822", "6821", "6825", "6828", "6829", "6839", "6850", "6851", "6860", "6869", "7110", "71102", "71106", "7119", "71109", "7304", "72806", "73007", "73018", "70319", "7301", "73025", "73026", "73029", "7302", "73097", "73098", "73099", "7309", "59010", "59018", "5901", "5902", "5950", "5970", "5990", "6010", "6040", "6049", "6110", "6140", "6143", "6163", "6164", "6340", "6390", "6466", "6709", "6751";
ICD-10 code: "A00", "A01", "A02", "A020", "A021", "A022", "A028", "A029", "A03", "A030", "A031", "A032", "A033", "A038", "A039", "A04", "A040", "A041", "A042", "A043", "A044", "A045", "A046", "A047", "A048", "A049", "A05", "A050", "A051", "A052", "A053", "A054", "A058", "A059", "A06", "A060", "A061", "A062", "A063", "A064", "A065", "A066", "A067", "A068", "A069", "A07", "A080", "A081", "A082", "A083", "A084", "A085", "A09", "A15", "A16", "A17", "A170", "A171", "A178", "A179", "A18", "A19", "A20", "A200", "A201", "A202", "A203", "A207", "A208", "A209", "A21", "A210", "A211", "A212", "A213", "A217", "A218", "A219", "A22", "A220", "A221", "A222", "A227", "A228", "A229", "A23", "A230", "A231", "A232", "A233", "A238", "A239", "A24", "A240", "A241", "A242", "A243", "A25", "A250", "A251", "A259", "A26", "A260", "A267", "A268", "A269", "A27", "A28", "A280", "A281", "A282", "A288", "A289", "A30", "A31", "A32", "A320", "A321", "A327", "A328", "A329", "A33", "A34", "A35", "A36", "A37", "A38", "A39", "A390", "A391", "A392", "A393", "A394", "A395", "A398", "A399", "A40", "A400", "A401", "A402", "A403", "A408", "A409", "A41", "A410", "A411", "A412", "A413", "A414", "A415", "A418", "A419", "A42", "A420", "A421", "A422", "A427", "A428", "A429", "A43", "A430", "A431", "A438", "A439", "A44", "A440", "A441", "A448", "A449", "A46", "A480", "A481", "A482", "A483", "A484", "A488", "A49", "A490", "A491", "A492", "A493", "A498", "A499", "A50", "A500", "A501", "A502", "A503", "A504", "A505", "A506", "A507", "A509", "A51", "A510", "A511", "A512", "A513", "A514", "A515", "A519", "A52", "A520", "A521", "A522", "A523", "A527", "A528", "A529", "A53", "A54", "A540", "A541", "A542", "A543", "A544", "A545", "A546", "A548", "A549", "A55", "A560", "A561", "A562", "A563", "A564", "A568", "A57", "A58", "A59", "A60", "A630", "A638", "A64", "A65", "A66", "A67", "A68", "A69", "A690", "A691", "A692", "A698", "A699", "A70", "A71", "A740", "A748", "A749", "A75", "A750", "A751", "A752", "A753", "A759", "A77", "A770", "A771", "A772", "A773", "A778", "A779", "A78", "A79", "A790", "A791", "A798", "A799", "A80", "A811", "A812", "A83", "A84", "A85", "A86", "A87", "A88", "A89", "A90", "A91", "A92", "A920", "A921", "A922", "A923", "A924", "A925", "A928", "A929", "A93", "A930", "A931", "A932", "A938", "A94", "A95", "A96", "A97", "A98", "A980", "A981", "A982", "A983", "A984", "A985", "A988", "A99", "B00", "B000", "B001", "B002", "B003", "B004", "B005", "B007", "B008", "B009", "B01", "B010", "B011", "B012", "B018", "B019", "B02", "B020", "B021", "B022", "B023", "B027", "B028", "B029", "B04", "B05", "B050", "B051", "B052", "B053", "B054", "B058", "B059", "B06", "B060", "B068", "B069", "B07", "B080", "B081", "B082", "B083", "B084", "B085", "B088", "B09", "B15", "B150", "B159", "B16", "B160", "B161", "B162", "B169", "B17", "B18", "B19", "B190", "B199", "B20", "B21", "B210", "B22", "B220", "B221", "B222", "B227", "B23", "B24", "B25", "B26", "B260", "B261", "B262", "B263", "B268", "B269", "B27", "B270", "B271", "B278", "B279", "B30", "B33", "B330", "B331", "B332", "B333", "B334", "B338", "B34", "B340", "B341", "B342", "B343", "B344", "B348", "B349", "B35", "B36", "B37", "B370", "B371", "B372", "B373", "B374", "B375", "B376", "B377", "B378", "B379", "B38", "B380", "B381", "B382", "B383", "B384", "B387", "B388", "B389", "B39", "B390", "B391", "B392", "B393", "B394", "B395", "B399", "B40", "B400", "B401", "B402", "B403", "B407", "B408", "B409", "B41", "B410", "B417", "B418", "B419", "B42", "B420", "B421", "B427", "B428", "B429", "B43", "B430", "B431", "B432", "B438", "B439", "B44", "B45", "B450", "B451", "B452", "B453", "B457", "B458", "B459", "B46", "B460", "B461", "B462", "B463", "B464", "B465", "B468", "B469", "B47", "B470", "B471", "B479", "B48", "B480", "B481", "B482", "B483", "B484", "B487", "B488", "B49", "B50", "B500", "B508", "B509", "B51", "B52", "B53", "B530", "B531", "B538", "B54", "B550", "B551", "B552", "B559", "B56", "B57", "B58", "B580", "B581", "B582", "B583", "B588", "B589", "B59", "B600", "B601", "B602", "B608", "B64", "B65", "B650", "B651", "B652", "B653", "B658", "B659", "B66", "B660", "B661", "B662", "B663", "B664", "B665", "B668", "B669", "B67", "B68", "B680", "B681", "B689", "B69", "B690", "B691", "B698", "B699", "B70", "B700", "B701", "B71", "B710", "B711", "B718", "B719", "B72", "B73", "B74", "B75", "B76", "B77", "B78", "B79", "B80", "B81", "B810", "B811", "B812", "B813", "B814", "B818", "B82", "B820", "B829", "B83", "B830", "B831", "B832", "B833", "B834", "B838", "B839", "B85", "B86", "B87", "B88", "B880", "B881", "B882", "B883", "B888", "B889", "B89", "B95", "B960", "B961", "B962", "B963", "B964", "B965", "B966", "B967", "B968", "B97", "B970", "B971", "B972", "B973", "B974", "B975", "B976", "B977", "B978", "B980", "B981", "B99", "C46", "D733", "E321", "G00", "G000", "G001", "G002", "G003", "G008", "G009", "G01", "G020", "G021", "G028", "G03", "G041", "G042", "G050", "G051", "G052", "G06", "G07", "H00", "H010", "H050", "H061", "H100", "H105", "H130", "H190", "H191", "H192", "H220", "H320", "H440", "H600", "H601", "H602", "H603", "H620", "H621", "H622", "H623", "H660", "H670", "H671", "H700", "H750", "I301", "I320", "I321", "I330", "I400", "I410", "I411", "I412", "I430", "I520", "I521", "I681", "J010", "J02", "J020", "J028", "J029", "J03", "J030", "J038", "J039", "J04", "J051", "J09", "J10", "J11", "J12", "J13",

© 2023 Tang R et al. *JAMA Network Open.*

"J14", "J15", "J150", "J151", "J152", "J153", "J154", "J155", "J156", "J157", "J158", "J159", "J16", "J160", "J168", "J170", "J171", "J172", "J173", "J178", "J18", "J20", "J200", "J201", "J202", "J203", "J204", "J205", "J206", "J207", "J208", "J209", "J21", "J210", "J211", "J218", "J219", "J22", "J36", "J390", "J391", "J851", "J852", "J853", "J86", "K02", "K040", "K044", "K045", "K046", "K047", "K050", "K052", "K053", "K054", "K113", "K122", "K230", "K231", "K35", "K570", "K572", "K574", "K578", "K61", "K630", "K650", "K670", "K671", "K672", "K673", "K678", "K750", "K751", "K770", "L00", "L01", "L02", "L03", "L04", "L05", "L08", "L701", "M00", "M000", "M001", "M002", "M008", "M009", "M010", "M011", "M012", "M013", "M014", "M015", "M016", "M018", "M462", "M463", "M465", "M490", "M491", "M492", "M493", "M600", "M630", "M631", "M632", "M650", "M651", "M710", "M711", "M726", "M730", "M731", "M86", "M860", "M861", "M862", "M863", "M864", "M865", "M866", "M868", "M869", "N080", "N10", "N136", "N151", "N160", "N290", "N291", "N300", "N340", "N390", "N410", "N412", "N45", "N61", "N700", "N710", "N730", "N733", "N74", "N740", "N741", "N742", "N743", "N744", "N748", "N751", "N760", "N764", "N770", "N771", "O030", "O035", "O040", "O045", "O050", "O055", "O060", "O065", "O070", "O075", "O080", "O230", "O231", "O232", "O233", "O234", "O235", "O239", "O753", "O85", "O86", "O910", "O911", "O98", "O980", "O981", "O982", "O983", "O984", "O985", "O986", "O987", "O988", "O989", "P23", "P230", "P231", "P232", "P233", "P234", "P235", "P236", "P238", "P239", "P350", "P351", "P352", "P353", "P36", "P360", "P361", "P362", "P363", "P364", "P365", "P368", "P369", "P37", "P370", "P371", "P372", "P373", "P374", "P375", "P378", "P379", "P38", "P39", "P390", "P391", "P392", "P393", "P394", "P398", "P399", "R572", "R650", "R651", "U04", "U06", "U071", "U072", "U10", "Z21".

History of immune diseases

Immune disease is defined as ICD-10 code: "J45", "J46", "M05", "M06", "M08", "K51", "E10", "I00"- "I02", "I05"- "I09", "L40", "K90.0", "K50", "M35.3", "G35", "J301"- "J304", "M79.0", "M79.0", "M79.0", "M07", "E05.0", "M06.3", "M45", "M31", "D86", "L43", "M35.0", "L93", "M32", "D69.3", "K74.3", "M60", "G61.0", "G70.0", "L10"- "L14", "K75.4".

History of nephrotoxic drugs use

Nephrotoxic drugs use is defined as taking medications that have been linked to kidney damage in UKB (ibuprofen: filed 6154: 2; hydrochlorothiazide: field 20003: 1140860332, 1140860404, 1140860422, 1140860562, 1140860738, 1140860764, 1140860790, 1140864950, 1140864952, 1140866162, 1140926778, 1141151016, 1141172682, 1141187788, 1141201038; lisinopril: field 20003: 1140860696,1140864952; vancomycin: field 20003: 1140873850; gentamicin: field 20003: 1140853854, 1140873574, 1140875582, 1140882842, 1141157498, 1141190776).

© 2023 Tang R et al. *JAMA Network Open.*

eTable1. Definition of Diet Score in UK Biobank

Diet components	Field IDs	Healthy diet score
Fruits	1309 (fresh fruit/day intake)<br>1319 (dried fruit/day intake)	1 for ≥3 pieces/day<br>0 for <3 pieces/day
Vegetables	1289 (cooked vegetables/day intake)<br>1299 (salad/raw vegetable intake)	1 for ≥4 tablespoons/day<br>0 for <4 tablespoons/day
Fish	1329 (oily fish intake)<br>1339 (non-oily fish intake)	1 for ≥2 times/week<br>0 for <2 times/week
Unprocessed meat	1369 (beef intake)<br>1379 (lamb/mutton intake)<br>1389 (pork intake)	1 for <2 times/week<br>0 for ≥2 times/week
Processed meat	1349 (processed meat intake)	1 for <2 times/week<br>0 for ≥2 times/week

The sum of five diet components is the overall diet score, range from 0-5. A higher score indicates a better diet score.

© 2023 Tang R et al. *JAMA Network Open.*

eTable2. Proportional Hazard Assumption P-Values and Kaplan-Meier Plots

Variables	Log rank test *P* value	Time interaction *P* value	Log-log plot
**Age**	<0.001	0.07	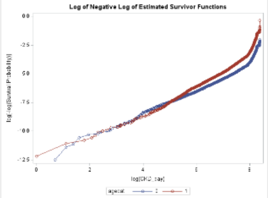
**Sex**	<0.001	0.06	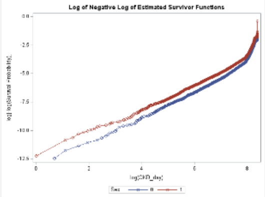
**Ethnicity**	<0.001	0.25	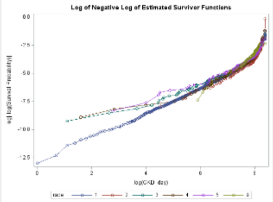
**BMI**	<0.001	0.43	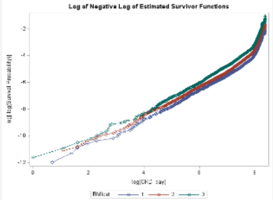
**Townsend deprivation index**	<0.001	0.65	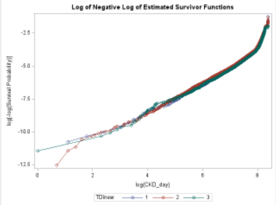

© 2023 Tang R et al. *JAMA Network Open.*

**Smoking**	<0.001	0.25	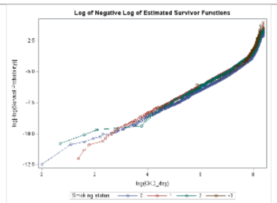
**Drinking**	<0.001	0.32	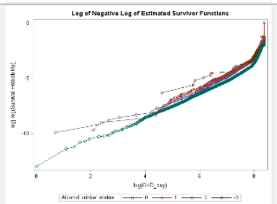
**Regular physical activity**	<0.001	0.26	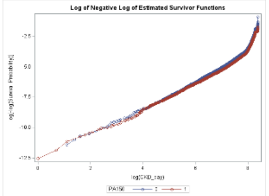
**High cholesterol**	<0.001	0.09	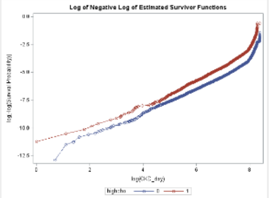
**Cardiovascular disease**	<0.001	0.43	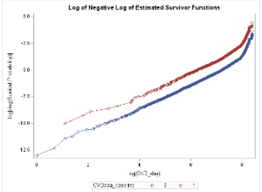
**Diabetes**	<0.001	0.90	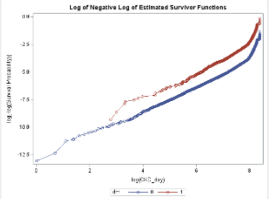

© 2023 Tang R et al. *JAMA Network Open.*

eTable3. Sensitivity Analyses for Association Between Self-Reported Frequency of Adding Salt to Food and Hazard of Chronic Kidney Disease

Models	Frequency of adding salt to food				
	Never/rarely	Sometimes	Usually	Always	*P*-trend
Excluding participants who have hypertension at baseline [a]	1(reference)	1.04 (0.99, 1.08)	1.07 (1.02, 1.13)	1.15 (1.07, 1.25)	<0.001
Excluding participants who have diabetes or CVD at baseline [b]	1(reference)	1.03 (0.99, 1.06)	1.06 (1.01, 1.10)	1.14 (1.07, 1.21)	<0.001
Excluding participants who develop CKD <12 months after recruitment [c]	1(reference)	1.03 (1.00, 1.06)	1.06 (1.01, 1.10)	1.11 (1.05, 1.18)	<0.001
Excluding participants who changed their diet in last 5 years [d]	1(reference)	1.01 (0.97, 1.06)	1.05 (1.00, 1.12)	1.15 (1.06, 1.24)	0.001
Multivariable adjusted + Diet score [e]	1(reference)	1.02 (0.99, 1.05)	1.04 (1.00, 1.09)	1.09 (1.02, 1.16)	0.003

Models were adjusted for sex, age, race, BMI, Townsend deprivation Index, smoking, drinking, regular physical activity, high cholesterol.
[a], A total of 345,661 participants were available.
[b], A total of 434,819 participants were available.
[c], A total of 462,577 participants were available.
[d], A total of 284,095 participants were available.
[e], A total of 448,932 participants were available.
BMI: body mass index; CKD: chronic kidney disease; CVD: cardiovascular disease.

© 2023 Tang R et al. *JAMA Network Open.*

eTable4. The Association Between the Self-Reported Frequency of Adding Salt to Foods and Hazard of Chronic Kidney Disease by eGFR Levels (After Excluding Ever Smokers)

	Frequency of adding salt to food					*P* for interaction
	Never/rarely	Sometimes	Usually	Always	*P*-trend	
eGFR						0.69
≥90 mL/min per 1.73 m^2	1(reference)	0.97 (0.90-1.04)	1.05 (0.95-1.17)	1.23 (1.06-1.43)	0.07	
60-90 mL/min per 1.73 m^2	1(reference)	1.05 (0.99-1.11)	1.07 (0.99-1.16)	1.07 (0.94-1.21)	0.04	

Models were adjusted for sex, age, race, BMI, Townsend deprivation Index, smoking, drinking, regular physical activity, high cholesterol.
BMI: body mass index; eGFR: estimated glomerular filtration rate.
eGFR, estimated glomerular filtration rate.

eReference

1. Lees JS, Welsh CE, Celis-Morales CA, et al. Glomerular filtration rate by differing measures, albuminuria and prediction of cardiovascular disease, mortality and end-stage kidney disease. *NATURE MEDICINE*. 2019;25(11):1753.

© 2023 Tang R et al. *JAMA Network Open.*
