# Supplementary material for: Self-Reported Frequency of Adding Salt to Food and Risk of Incident Chronic Kidney Disease
Source: JAMA Netw Open. 2023 Dec 28;6(12):e2349930. doi: 10.1001/jamanetworkopen.2023.49930 (PMC10755616; doi:10.1001/jamanetworkopen.2023.49930)
Supplement: Supplement 2. — Data Sharing Statement [file jamanetwopen-e2349930-s002.pdf]

Data Sharing Statement

Tang. Self-Reported Frequency of Adding Salt to Food and Risk of Incident Chronic Kidney Disease. *JAMA Netw Open*. Published December 28, 2023.
doi:10.1001/jamanetworkopen.2023.49930

Data
Data available: No

Additional Information
Explanation for why data not available: This study has been conducted using the UK Biobank Resource, approved project number 29256. The UK Biobank will make the source data available to all bona fide researchers for all types of health-related research that is in the public interest, without preferential or exclusive access for any persons. All researchers will be subject to the same application process and approval criteria as specified by UK Biobank. For more details on the access procedure, see the UK Biobank website: http://www.ukbiobank.ac.uk/register-apply.
